# Presence and Comprehensiveness of Antibullying Policies for Faculty at US Medical Schools

**DOI:** 10.1001/jamanetworkopen.2022.28673

**Published:** 2022-08-23

**Authors:** Maya S. Iyer, Yujung Choi, Cherri Hobgood

**Affiliations:** 1 Nationwide Children’s Hospital, Columbus, Ohio; 2 The Ohio State University College of Medicine, Columbus; 3 Duke University, Durham, North Carolina; 4 Indiana University School of Medicine, Indianapolis

## Abstract

This cross-sectional study assesses the presence and comprehensiveness of antibullying policies at top US medical schools.

## Introduction

Bullying, a severe form of mistreatment, occurs in the medical setting when a power differential allows offenders to consciously target individuals through persistent negative actions to impede the education or career of the target.^1^ Targets may experience burnout, depression, anxiety, decreased productivity, and job attrition.^1^ In medical settings, being bullied is associated with adverse clinical events, impacting the quality of patient care.^2^

While medical trainees are protected by policies of the Accreditation Council of Graduate Medical Education, bullying protection policies for practicing physicians are institution-dependent and lack standardization. To address this, the American Medical Association (AMA) adopted an antibullying policy in 2020 that identified the need for all health care environments to “establish policies to prevent and address bullying” and provided criteria for comprehensiveness.^3^ The purpose of this study is to (1) determine whether medical schools have defined faculty antibullying policies and (2) evaluate policy comprehensiveness using the AMA’s criteria.^3^

## Methods

This cross-sectional study was deemed exempt from review and informed consent by the Nationwide Children’s Hospital institutional review board because it was not deemed human participants research. This study is reported following the Strengthening the Reporting of Observational Studies in Epidemiology (STROBE) reporting guideline.

In March to June 2022, we conducted a cross-sectional study sampling schools from the US News and World Report’s top medical schools^4^ for research and for primary care to generate 3 cohorts: the top 25 schools from research and primary care rankings, 25 schools randomly selected from research rankings, and 25 schools randomly selected from primary care rankings. We characterized schools as having: (1) antibullying policies, (2) antiharassment policies mentioning bullying, (3) antiharassment policies without mentioning bullying, or (4) no policies. We assessed policy comprehensiveness by evaluating whether the policy includes faculty members, describes institution’s commitment to providing a safe and healthy workplace, defines bullying (expected and prohibited behaviors), discusses roles and responsibilities for employees, outlines steps to take when experiencing bullying, provides a confidential reporting procedures, prohibits retaliation and ensures privacy and confidentiality, and documents training requirements, per criteria provided by the AMA.^3^

We searched medical schools’ websites using the following keywords and 16 combinations thereof: *faculty*, *policy*, *bylaws*, *handbook*, *governance*, *bullying*, *discrimination*, *harassment*, and *mistreatment*. For schools without identifiable policies, we searched the term *antiharassment* and then *antibullying* to identify antibullying language embedded within antiharassment policies. If these searches yielded no results or required a login, we categorized that school as having no policy. We used descriptive statistics in aggregate to assess the presence or absence of a dichotomous variable. We did not compare groups because our aim was to probe the overall status of policies addressing bullying. We mitigated bias through randomly selecting schools, a uniform search strategy, and using a dichotomous variable as a primary outcome measure.

## Results

We identified 91 medical schools. Among these, 4 schools had antibullying policies with reporting procedures, but no school met all comprehensiveness criteria (Table). Among the 87 medical schools without antibullying policies, 60 had antiharassment policies; of these, 10 schools mentioned bullying and included reporting procedures. A total of 26 schools lacked antibullying and antiharassment policies. There were 5 medical schools that required login credentials to access policies, and 1 school had a broken webpage link.

**Table.** Comprehensiveness of Identified Antibullying Policies

Medical school	Policy criteria met							
	Includes faculty members	States commitment to safe and healthy workplace	Defines bullying (expected/prohibited behaviors)	Discusses roles and responsibilities of employees^a^	Outlines steps to take when experiencing bullying	Includes confidential reporting procedures with specific contact information	Prohibits retaliation, ensures privacy and confidentiality	Documents training requirements
University of California, Davis^b^	Yes	Yes	Yes	Yes	No	Yes	Yes	No
University of Colorado^c^	Yes	Yes	Yes	Yes	Yes	No	Yes	No
University of Wisconsin-Madison^d^	Yes	Yes	Yes	Yes	Yes	No	No	No
University of Pikeville College of Osteopathic Medicine^e^	Yes	Yes	Yes	No	Yes	No	Yes	No

^a^ Roles and responsibilities may include individual conduct themselves with respect for the rights and welfare of others in the workplace and not engage in bullying and/or abusive conduct; report instances of bullying and/or abusive conduct that they are subjected to and/or witness occurring to someone else; organizations should foster a respectful, fair, safe, equitable, and secure working environment; consult with the appropriate university offices about allegations of bullying and/or abusive conduct; and/or review allegations of bullying and take appropriate corrective or disciplinary action on substantiated allegations after consultation with the appropriate offices has taken place.

^b^ Accessed from https://ucdavispolicy.ellucid.com/documents/view/105/active and https://ucanr.edu/sites/anrstaff/files/348069.pdf.

^c^ Accessed from https://www.cu.edu/ope/aps/5059.

^d^ Accessed from https://hr.wisc.edu/hib/ and https://intranet.med.wisc.edu/building-community/shared-guidelines-for-professional-conduct/reporting-concerns-smph-shared-guidelines-for-professional-conduct/#informal-approaches-faculty.

^e^ Accessed from https://www.upike.edu/wp-content/uploads/2021/11/Employee-Handbook-2020-21-9.21.2021.pdf#page=71.

## Discussion

This cross-sectional study found that among a random sampling of top-ranked US medical schools, most medical schools lacked antibullying policies. Without clear antibullying policies, the identification of bullying behaviors is ambiguous, reporting is low-to-absent, and bullies have nonstandardized repercussions.^5^ This signals a culture tolerant of bullying and an environment in which bullying can be perpetuated.^1^

Most medical schools did have written antiharassment policies; however, these policies are designed to support protected class individuals. Targets of bullying may not meet protected class criteria; therefore, these policies provide little recourse.^6^ This underscores the importance of policies with specific language naming and defining bullying, clear roles and responsibilities for faculty and administrators, and consequences for violators. Limitations of our study included not evaluating all medical US medical schools and being unable to access polices requiring institutional log-in credentials or intranet. To support the eradication of bullying, clear, comprehensive institutional antibullying policies are needed.
